# Supplementary material for: Resolving the full spectrum of human genome variation using Linked-Reads
Source: Genome Res. 2019 Apr;29(4):635–45. doi: 10.1101/gr.234443.118 (PMC6442396; doi:10.1101/gr.234443.118)
Supplement: Supplemental Material [file supp_gr.234443.118_Supplemental_longranger-2.2.zip › longranger-2.2.tar.bz2!/lr2.2-public/lib/stan/cmdstan/stan/doc/api/html/dir_b1c6471129ce395961fd25e68f861508.html]

Stan: src/stan Directory Reference


|  |  |
| --- | --- |
|  | Stan  2.5.0  probability, sampling & optimization |


- Main Page
- Related Pages
- Namespaces
- Classes
- Files

All Classes Namespaces Files Functions Variables Typedefs Enumerations Enumerator Friends Macros Pages

- src
- stan

stan Directory Reference

|  |  |
| --- | --- |
| Directories | |
| directory | agrad |
|  | |
| directory | command |
|  | |
| directory | common |
|  | |
| directory | gm |
|  | |
| directory | io |
|  | |
| directory | math |
|  | |
| directory | mcmc |
|  | |
| directory | memory |
|  | |
| directory | meta |
|  | |
| directory | model |
|  | |
| directory | optimization |
|  | |
| directory | prob |
|  | |

|  |  |
| --- | --- |
| Files | |
| file | agrad.hpp [code] |
|  | |
| file | common.hpp [code] |
|  | |
| file | gm.hpp [code] |
|  | |
| file | io.hpp [code] |
|  | |
| file | math.hpp [code] |
|  | |
| file | mcmc.hpp [code] |
|  | |
| file | memory.hpp [code] |
|  | |
| file | meta.hpp [code] |
|  | |
| file | model.hpp [code] |
|  | |
| file | prob.hpp [code] |
|  | |
| file | version.hpp [code] |
|  | |


---

[ Stan Home Page ]

*© 2011–2014,
Stan Development Team.*
